# Serum Adropin Levels in Patients on Hemodialysis

**DOI:** 10.3390/life11040337

**Published:** 2021-04-11

**Authors:** Dijana Boric-Skaro, Maja Mizdrak, Mirko Luketin, Dinko Martinovic, Daria Tokic, Marino Vilovic, Daniela Supe-Domic, Tina Ticinovic Kurir, Josko Bozic

**Affiliations:** 1 Department of Nephrology and Dialysis, University Hospital of Split, 21000 Split, Croatia; dborskaro@kbsplit.hr (D.B.-S.); mmizdrak@mefst.hr (M.M.); mluketin@kbsplit.hr (M.L.); 2 Department of Pathophysiology, University of Split School of Medicine, 21000 Split, Croatia; dinko.martinovic@mefst.hr (D.M.); marino.vilovic@mefst.hr (M.V.); tticinov@mefst.hr (T.T.K.); 3 Department of Anesthesiology and Intensive Care, University Hospital of Split, 21000 Split, Croatia; dtokic@kbsplit.hr; 4 Department of Health Studies, University of Split, 21000 Split, Croatia; daniela.supe.domic@ozs.unist.hr; 5 Department of Endocrinology, University Hospital of Split, 21000 Split, Croatia

**Keywords:** adropin, hemodialysis, malnutrition-inflammation score, dialysis malnutrition score, body fat percentage, lipid profile

## Abstract

Adropin is a novel pleotropic peptide involved in energy homeostasis, with possible contribution to cardiovascular protection through production of nitric oxide and subsequent blood pressure regulation. Given that patients undergoing hemodialysis (HD) are related with high cardiovascular risk, hyperlipidemia, chronic low-grade inflammation, and malnutrition the aim of our study was to investigate serum adropin levels in HD patients to evaluate possible associations with nutritional status and other relevant clinical and laboratory parameters. The study included 70 patients on HD and 60 healthy controls. Serum adropin levels were determined by an enzyme-linked immunosorbent assay in a commercially available diagnostic kit. Serum adropin levels were significantly lower in the HD group compared to the control group (2.20 ± 0.72 vs. 4.05 ± 0.93 ng/mL, *p* < 0.001). Moreover, there was a significant negative correlation with malnutrition-inflammation score (r = −0.476, *p* < 0.001), dialysis malnutrition score (r = −0.350, *p* = 0.003), HD duration (r = −0.305, *p* = 0.010), and high sensitivity C-reactive protein (hsCRP) (r = −0.646, *p* < 0.001). Additionally, there was a significant negative correlation between adropin levels and pre-dialysis systolic (r = −0.301, *p* = 0.011) and diastolic blood pressure (r = −0.299, *p* = 0.011). These results are implying that adropin is potentially involved in the pathophysiological mechanisms of chronic kidney disease (CKD)/HD and its complications. However, future larger scale longitudinal studies need to further address it.

## 1. Introduction

Hemodialysis (HD) is an artificial procedure of removing excess fluids, minerals, and toxins from the blood of patients who have an impaired renal function. HD is one of the most used renal replacement therapies and a life-depending method for patients with chronic kidney disease (CKD), the procedure is associated with a high cardiovascular risk, morbidity, and mortality [1,2].

Adropin is a novel pleotropic peptide which is encoded by the *ENHO* gene whose expression was found in the liver and brain, but its presence was also established in the muscle, heart, pancreas, and kidneys [3,4]. However, studies have showed that adropin has a wide range of diverse effects, among which the most prominent one is maintaining energy homeostasis through glucose and lipid metabolism regulation [5,6,7]. In a study by Akcilar et al. [8] it was presented that mice with dietary induced obesity have a higher glucose tolerance and reduced insulin resistance after peritoneal treatment with adropin. Another study conducted on obese patients and healthy controls showed that serum adropin levels were lower in participants with obesity and insulin resistance whereas lower body mass index (BMI) was linked with the rise of serum adropin levels [9]. Additionally, a recent study showed that adropin treatment downregulated the expression of gluconeogenic regulatory enzymes in the liver which consequently led to inhibition of hepatic glucose production and improved hepatic insulin sensitivity [10].

Growing evidence also suggest that adropin possibly plays a significant role in the cardiovascular system. Several studies pointed to a possible association between adropin and blood pressure, neovascularization, and vascular protection, while it was indicated that adropin induces production of nitric oxide by regulating endothelial nitric oxide synthase and vascular endothelial growth factor receptor 2 (VEGF2) [11,12,13]. Moreover, it was found that patients with coronary heart disease have low serum adropin levels compared to the healthy controls and Yu et al. [14] also showed that a decrease of serum adropin levels in patients with coronary artery disease could predict the incidence of acute myocardial infarction. Furthermore, the most recent studies are linking adropin with chronic inflammatory states and are proposing a possible immunomodulatory effect. It has been showed that patients with obstructive sleep apnea [15], inflammatory bowel diseases [16], polycystic ovary syndrome [17] and diabetes [18] have significantly lower serum levels of adropin. The perceived link is still not understood but it is possible that endothelial dysfunction is the main driver for the serum adropin levels reduction in these disorders.

Since CKD/HD settings are associated with a high cardiovascular risk [1,19,20], hyperlipidemia [21], hyperinsulinemia [22], chronic low-grade inflammation [23], and malnutrition [24], it is reasonable to presume a potential link with adropin. A recent study conducted on HD patients determined that serum adropin levels are lower in HD patients compared to healthy controls [25]. However, two other studies did not find any significant difference between HD patients and healthy controls [26,27]. Since these results are contradictive and none of these studies investigated the possible connection between adropin and nutritional status of the HD patients, the evidence regarding the possible link between adropin and CKD/HD patients is still scarce.

So, the aim of this study was to further investigate serum adropin levels in HD patients and to evaluate possible associations with nutritional status and other relevant clinical and laboratory parameters.

## 2. Materials and Methods

### 2.1. Study Design and Subject Inclusion Criteria

This cross-sectional study was conducted at the University Hospital of Split in the period from 1 January to 1 April 2019. The study was performed according to the ethical principles of the Declaration of Helsinki from 2013, and approved by the Ethics Committee of the University Hospital of Split. All subjects signed a written informed consent to participate.

The study included 70 patients who are undergoing HD at the Department of Nephrology and Dialysis University Hospital of Split and 60 healthy controls. Control subjects were volunteers recruited through the University Hospital of Split and they did not receive any compensation.

The inclusion criteria were patients above 18 years of age; undergoing HD more than one year on an intermittent program; stability during HD sessions; received dialysis dose (Kt/V) ≥ 1.2; body mass index (BMI) between 18.5 and 35 kg/m^2^; patients with stable weight; HbA1c < 9%. Exclusion criteria were that in the period of 3 months prior to the study potential participants had a history of: stroke and myocardial infarction; uncontrolled hypertension; alcoholism; autoimmune diseases; malignancies; liver diseases; hypoglycemia episodes; and receiving corticosteroid therapy. The patients undergoing HD received standard care and therapy. All included subjects underwent detailed physical examination and review of medical history. Furthermore, they were interviewed for drugs, tobacco, and alcohol consumption.

Control subjects were further screened for possible renal diseases. They underwent a detailed physical examination, screening and medical history reviewing. All potential participants who had a confirmed or suspected renal disease were excluded from the study. Renal disease was considered absent if the eGFR (CKD-EPI (Chronic Kidney Disease Epidemiology Collaboration) creatinine equation) was >60 mL/min/1.73 m^2^.

### 2.2. Hemodialysis Sessions

All CKD patients were undergoing intermittent HD, with bicarbonate dialysate at a flow rate of 375–450 mL/min and low molecular weight heparin (LMWH) as standard anticoagulation using low flux (ultrafiltration rate <20 mL/mmHg/h) polysulfone membrane dialyzers FX8 and FX60 (Fresenius, Bad Homburg, Germany) with a blood flow rate of 250–300 mL/min. Temperature of dialysate was maintained at 36–37 °C and the dialysis bath consisted of bicarbonate 32–35 mmol/L, sodium 138–145 mmol/L, potassium 2 mmol/L, and calcium 1.25–1.5 mmol/L. Ultrafiltration was measured volumetrically on the dialysis machine.

### 2.3. Laboratory Analysis

Blood samples were taken after 12-h fastening and handled according to standard laboratory practice, by an experienced blinded medical biochemist. The samples for analyses of serum adropin levels were centrifuged and stored at −80 °C for further analyses, while the hematological and biochemical parameters were analyzed on the same day using the standard laboratory procedures. In HD patients, the blood sampling was performed right before the dialysis session.

Serum adropin levels were determined using the dual enzyme-linked immunosorbent assay (ELISA) of human adropin (Phoenix Pharmaceuticals, Burlingame, CA, USA), according to the manufacturer’s instructions. Calibrations were double measured, whereas optical density (OD) values were in accordance with predefined OD values stated in manufacturer instructions and coefficient of variability (CV) of paired calibrations were <15%. The linear range of the assay was 0.3–8.2 ng/mL and sensitivity was 0.3 ng/mL, while the CV within the probe was less than 10%.

### 2.4. Anthropometric Measurements and Clinical Assessment

Body height and mass were determined using a medical scale with built-in heights (Seca, Birmingham, UK). Body mass index (BMI) was calculated using the formula = body mass/height2 (kg/m^2^). In HD patients, the measurements were performed after the HD session.

Skinfold thickness was measured using the professional skinfold caliper (Gima S.p.A., Gessate, MI, Italy) at three different locations, depending on the gender of the subject. For males, measurement sites were chest, abdomen, and thigh, while for females they were triceps, suprailiac crest, and thigh. The precise locations were marked with a washable marker before the measurements, while for achieving consistency, all skinfold measurements were taken at on the right side of a relaxed body. Mean of three measurements for each skinfold was calculated to achieve an accurate value. Skinfold thicknesses at the designated locations were later used in Jackson–Pollock formulas for obtaining the percentage of the subject’s body fat [28]. In HD patients, the measurements were performed after the HD session.

Bioimpedance was measured with a Body Composition Monitor (Fresenius, Bad Homburg, Germany). The measurements required the patient to lie on a flat surface for 2 min. Whole body bioimpedance measurements were conducted on one arm and one leg of the patients. The electrodes were placed on the dorsal side of the hand and over an imaginary line of the wrist. They are placed on the foot on the dorsal side of the foot and over the imaginary ankle line joint. After placing the electrodes, it is necessary to enter the age, gender, body weight and height of the patient into the device. The measurement was performed prior to the HD session.

Blood pressure measurements were conducted before and right after the dialysis session. Three measurements were performed, and the mean was calculated to achieve a more precise blood pressure value. 

### 2.5. Nutrition and Inflammation Assessment Scores

Two validated scoring systems were used to evaluate the nutritional and inflammatory status of the patients who are undergoing HD. The evaluations were performed by the same trained physician within 30 min before HD sessions.

Malnutrition Inflammation Score is a grading system used for assessing the presence and evaluating the degree of nutritional deficit and inflammatory conditions in patients who undergo dialysis. It is based on 10 components, with each having a score between 0—normal and 3—severely abnormal. The sum of all ten Malnutrition-Inflammation Score (MIS) components ranges from 0 to 30, with the higher score indicating a more severe degree of malnutrition and inflammation [29].

Dialysis Malnutrition Score is a grading system used for assessment of malnutrition in patients undergoing dialysis. It is based on 7 components, with each one having a score between 1—normal and 5—severely abnormal. The sum of all seven Dialysis Malnutrition Score (DMS) components ranges from 7 to 35; with the higher score indicating a more severe degree of malnutrition [30].

### 2.6. Statistical Analysis

All statistical analyses were performed using statistical software MedCalc (MedCalc Software, Ostend, Belgium, version 17.4.1). Sample size analysis was conducted using the data from a pilot study on 12 subjects from the HD population and 12 matched control subjects. The value of serum adropin, which was the main result of the study, was used for the calculation. The mean serum adropin levels were 2.89 ± 0.71 ng/mL in the HD group and 3.71 ± 0.96 ng/mL in the control group. With type I error of 0.05, and the power of 90%, the required sample size was 23 participants per group.

Quantitative data was expressed as mean ± standard deviation or median and interquartile range, while qualitative data was expressed as whole number and percentage. For estimating the normality of data distribution Kolmogorov–Smirnov test was used. Comparison of quantitative variables was performed by Student t-test for independent samples or Mann–Whitney U test, while comparison of qualitative variables was performed by Chi-squared test. Pearson’s correlation or Spearman’s correlation were used to calculate the correlation between laboratory, anthropometric and clinical parameters with serum adropin levels. Additionally, multiple linear regression analysis of independent predictors for adropin levels was performed, with reporting the corresponding p values with unstandardized β-coefficients, standard error, and t-values. The level of statistical significance was set at *p* < 0.05.

## 3. Results

### 3.1. Baseline Characteristics of the Study Population

There were no statistically significant differences between the HD group and the control group regarding gender, age, blood pressure and anthropometric measures. In the HD group, the median DMS was 13 (10–16) and the median MIS was 6 (4–9) (Table 1). There were 27 (38.5%) patients with hypertension in the HD group. As therapy 7 patients were treated with ACE inhibitors, 17 with calcium channel blockers, 21 with beta blockers, 12 with diuretics, 12 with moxonidin, 2 with urapidil, and 1 with minoxidil.

### 3.2. Laboratory Parameters of the Study Population

The HD group had significantly higher levels of urea (*p* < 0.001), creatinine (*p* < 0.001) and high sensitivity C-reactive protein (hsCRP) (*p* < 0.001), while the control group had significantly higher levels of hemoglobin (*p* < 0.001), total proteins (*p* < 0.001), albumins (*p* = 0.017), and HDL cholesterol (*p* = 0.005). There were no statistically significant differences between the HD group and the control group regarding the other parameters (Table 2).

### 3.3. Serum Adropin Levels

**Figure 1:**
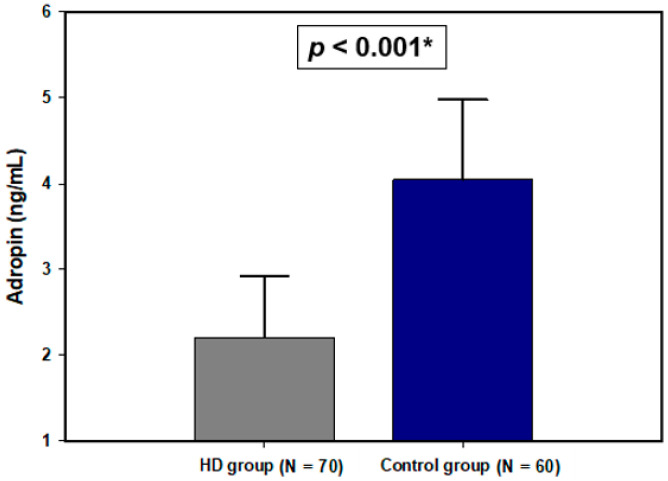
Plasma adropin levels in the HD group and the control group. Data are presented as mean ± standard deviation. * *t*-test for independent samples.

Serum adropin levels were significantly lower in the HD group compared to the control group (2.20 ± 0.72 vs. 4.05 ± 0.93 ng/mL, *p* < 0.001) (Figure 1). Furthermore, there was a statistically significant difference in serum adropin levels after dividing the HD group into diabetic and non-diabetic and comparing them (2.42 ± 0.88 vs. 2.13 ± 0.64 ng/mL, *p* = 0.132).

### 3.4. Adropin Correlations with Laboratory, Anthropometric and Clinical Parameters

There was a statistically significant negative correlation between serum adropin levels and HD duration (r = −0.305, *p* = 0.010), hsCRP (r = −0.646, *p* < 0.001), DMS (r = −0.350, *p* = 0.003), and MIS (r = −0.476, *p* < 0.001). Moreover, there was a significant correlation between serum adropin levels and pre−dialysis systolic pressure (r = −0.301, *p* = 0.011) and pre−dialysis diastolic pressure (r = −0.299, *p* = 0.011) (Table 3).

**Figure 2:**
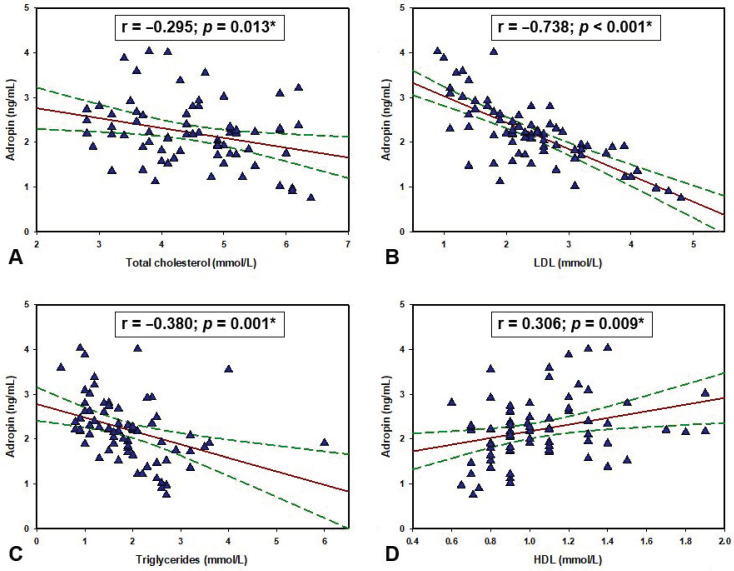
Correlation between adropin levels and (**A**) total cholesterol, (**B**) LDL, (**C**) triglycerides and (**D**) HDL levels in the hemodialysis population (N = 70). * Pearson’s correlation coefficient.

**Figure 3:**
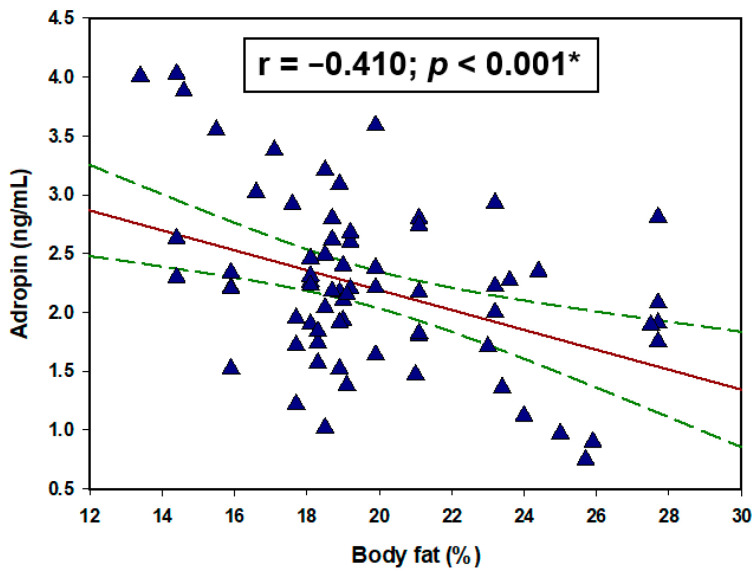
Correlation between serum adropin levels with body fat percentage (N = 70). * Pearson’s correlation coefficient.

Regarding the lipid profile of the HD group, there were significant negative correlations of adropin with total cholesterol (r = −0.295, *p* = 0.013), LDL (r = −0.738, *p* < 0.001), triglycerides (r = −0.380, *p* = 0.001) while there was a significant positive correlation with HDL (r = 0.306, *p* = 0.009) (Figure 2). Furthermore, there was a significant negative correlation between serum adropin levels and body fat percentage (r = −0.410, *p* < 0.001) (Figure 3). There were no statistically significant correlations with the other parameters (Table 3).

### 3.5. Multiple Linear Regression Analysis for Serum Adropin Levels

Multiple linear regression analysis showed that serum adropin levels as a dependent variable retained significant association with BMI (β ± SE, −0.027 ± 0.013, *p* = 0.049), HD duration (−0.031 ± 0.009, *p* = 0.001), hsCRP (−0.058 ± 0.019, *p* = 0.004), MIS (−0.310 ± 0.140, *p* = 0.030), and body fat percentage (−0.310 ± 0.140, *p* = 0.010) after model adjustment for age, gender, BMI, hsCRP, body fat percentage, MIS, DMS, and HD duration (Table 4).

## 4. Discussions

Our results showed that patients who are undergoing HD had significantly lower serum adropin levels compared to the healthy controls. This finding is in alignment with the outcomes of the study by Grzegorzewska et al. [25]. They showed that serum adropin levels were lower in the HD group compared to the controls and they further addressed this relationship after genotyping the study population for the ENHO gene where they found that participants who were major homozygotes for a certain polymorphism had significantly higher adropin levels and lower insulin resistance. Additionally, a study conducted on patients with diabetes mellitus type 2 who have developed diabetic nephropathy, showed that serum adropin levels were decreased in those patients [31].

Aforementioned findings suggest that adropin possibly plays a role in the complex CKD/HD pathophysiology. Several studies showed that adropin is an inductor of NO production through which it is associated with blood pressure regulation and vascular protection [11,32]. Given that our study found a significant negative correlation between adropin and pre-dialysis systolic and diastolic blood pressures, it could be hypothesized that this connection could be one of the links between CKD/HD and high cardiovascular risk. Study by Gu et al. [33] showed that adropin has a significant negative correlation with endothelin-1 (ET-1), a strong vasoconstrictor that is involved in hypertension pathogenesis. Moreover, recent studies are linking low adropin levels with the progression of endothelial dysfunction, another important factor in pathogenesis of cardiovascular diseases [34,35]. The findings of these aforementioned studies are suggesting that adropin is involved in pathogenesis of cardiovascular diseases associated with CKD/HD; however, why serum adropin levels are lower is still unclear.

An additional pathway which could explain adropin association with HD is the oxidative stress. Chen et al. [36] showed in their study conducted on mice with induced nonalcoholic steatohepatosis, that knockout of adropin substantially aggravated fibrosis and inflammation. In contrast, intraperitoneal administration of adropin suppressed proinflammatory mediators and stimulated expression of nuclear factor erythroid 2-related factor 2 (Nrf2), a major regulator of cellular resistance to oxidative stress [37]. Since oxidative stress is one of the pathophysiological hallmarks of HD due to retention of toxins, nutrition lacking antioxidants, loss of antioxidants during dialysis sessions and low-grade inflammation [38], it could be hypothesized that adropin with the induction of Nrf2 alleviates oxidative stress in those patients. Moreover, similarly to the other studies conducted on patients with chronic inflammatory conditions [17,39,40], we also found that serum adropin levels had a significant negative correlation with hsCRP. Given that all of those disorders also found lower serum adropin levels in comparison to healthy controls, it can be theorized that chronic inflammation decreases adropin levels. However, this needs to be further explored.

Contrary to our study, Kaluzna et al. [26,27] in their two studies did not find any significant difference of serum adropin levels between the dialysis patients and healthy controls. Moreover, they did not find any significant correlation between serum adropin levels and blood pressure. It is hard to explain this contradiction due to different methodologies of the studies. However, their patients were significantly younger, and it was showed that adropin decreases with age [35], a process which could be even more accelerated in CKD/HD settings. Moreover, they included patients who are undergoing peritoneal dialysis and they had significantly less male than female HD patients, both factors which could interfere with the results.

Another important finding of this study is the association between serum adropin levels and the laboratory lipid profile in the HD group. It was found that adropin has a negative correlation with triglycerides, LDL, and total cholesterols while it has a significant positive correlation with HDL cholesterol. The same correlations between lipids and adropin were found in several other studies [3,6,7,41]. Furthermore, a study conducted on HD patients with dyslipidemia showed that a certain variant of *ENHO* gene is associated with the hyper LDL cholesterolemic pattern of dyslipidemia, but contrary to expectations it was also associated with lower cardiovascular mortality [42]. Moreover, an animal study determined that intraperitoneal administration of adropin significantly reduces triglycerides, LDL and total cholesterol while also decreasing blood glucose, insulin levels, and homeostastic model assessment for insulin resistance (HOMA-IR) in rats with hyperlipidemia [8]. However, another animal study presented evidence that high cholesterol intake significantly suppresses *ENHO* gene expression [43]. Additionally, the same study showed that hypercholesterolemia resulting from a high cholesterol diet was not prevented in mice with adropin overexpression. These findings are pointing to the possibility that adropin is not involved in cholesterol uptake from the diet or its biosynthesis but rather that higher cholesterol uptake through a feedback mechanism inhibits its own production with demand while also regulating adropin expression. Several studies are showing that the dietary intake of certain macronutrients could be the main driver of adropin expression [6,7,44]. High fat diets have a stimulatory effect while diets rich in simple sugars are inhibitory on adropin’s expression. Nevertheless, with future studies we will get a better insight into adropin involvement in lipid homeostasis.

Our study also found a significant negative correlation between serum adropin levels and body fat percentage. Several studies showed that obesity has a negative correlation with adropin levels, and implied that higher body fat proportions possibly have a negative impact on circulating levels of adropin [44,45,46,47]. However, all of those studies were conducted on obese patients who also have insulin resistance, diabetes, or metabolic syndrome, all of which are known causes that inhibit adropin expression. In the study by Kumar et al. [6] it was found that adropin overexpressing mice were protected from body weight gain when they were fed with a high-fat diet. Moreover, offsetting of body weight gain was also accompanied with the decrease of the body fat percentage. However, the same study determined that adropin overexpressing mice, fed with a high-fat diet for three months, had similar body weight as the wild mice. This implies that adropin overexpression only delays the body weight gain, but it does not prevent it. Furthermore, study by Jasaszwili et al. [48] determined that adropin treatment suppresses lipogenic genes expression in adipose tissue of obese mice. All of these findings suggest that adropin could have a role as a lipolytic mediator, however it is still unclear why its expression is suppressed in higher body fat percentages. Since our study also found a significant negative correlation with the MIS and DMS scores we could hypothesize that adropin is not regulated only by the body’s composition but also via the nutritional status. It is well established that HD patients suffer from malnutrition due to metabolic and hormonal derangements, insulin deprivation, inflammation, inadequate nutrient intake, and adverse effects of renal replacement therapy [24]. Since adropin has shown a broad range of potential connections with the type of macronutrients in dietary intake, it is possible that the malnutrition and special nutrition in the HD settings is one of the causes involved in adropin downregulation. Overall, our multiple linear regression analysis showed that serum adropin levels as a dependent variable retained significant association with MIS, body fat, BMI, HD duration, and hsCRP.

This study had several limitations. It had a relatively small sample size, and it was conducted in a single center. Moreover, we were not able to completely eliminate all the possible confounding effects and the cross-sectional design of the study does not allow determination of any causal associations. One more potential limitation is that we evaluated serum adropin levels only before the HD session, so we do not know how does the HD session impact serum adropin levels.

In conclusion, this study showed that patients undergoing HD had significantly lower serum adropin levels compared to the control group. The possible association between CKD/HD settings and adropin were further implied with the negative correlation between serum adropin levels and clinical malnutrition indices and lipid profile. Our results are implying that adropin is somehow involved in the pathophysiological mechanisms and pathways of CKD/HD and its complications. However, future larger scale longitudinal studies need to address this relationship.

## Figures and Tables

**Table 1:** Baseline characteristics of the HD group and the control group.

Parameter	HD Group (N = 70)	Control Group (N = 60)	*p* *
Male gender (N, %)	28 (40)	24 (40)	0.857
Age (years)	69.2 ± 13.0	66.8 ± 15.8	0.349
Body weight (kg)	74.5 ± 16.1	78.9 ± 14.7	0.111
Body height (cm)	173.6 ± 10.1	176.7 ± 9.7	0.084
Body mass index (kg/m^2^)	24.4 ± 5.1	25.2 ± 4.2	0.299
Waist circumference (cm)	98.2 ± 12.72	100.25 ± 12.1	0.354
Hip circumference (cm)	104.2 ± 10.5	107.2 ± 9.1	0.086
Pre-dialysis systolic pressure (mmHg)	136.2 ± 26.9	-	-
Post-dialysis systolic pressure (mmHg)	122.9 ± 26.6	119.5 ± 8.0 ^†^	0.502
Pre-dialysis diastolic pressure (mmHg)	71.0 ± 16.3	-	-
Post-dialysis diastolic pressure (mmHg)	69.2 ± 15.1	71.7 ± 8.5 ^†^	0.257
Smoking (N, %)	20 (28.6)	13 (21.7)	0.484
Diabetes mellitus (N, %)	18 (25.7)	-	-
HbA1C (%)	6.89 ± 1.16	5.61 ± 0.34	<0.001
CKD duration (years)	12 (7–31)	-	-
HD duration (years)	4.5 (3–9)	-	-
Prior kidney transplantation (N, %)	8 (11.4)	-	-
Prior PD (N, %)	5 (7.1)	-	-
Urine output (mL)	200 (0–1000)	-	-
DMS	13 (10–16)	-	-
MIS	6 (4–9)	-	-
Body fat percentage (%)	22.8 ± 7.7	21.6 ± 6.6	0.359
Dry body weight (kg) ^‡^	72.79 ± 15.64	-	-
IDWG (kg)	2.25 ± 0.84	-	-

Abbreviations: HD—hemodialysis; CKD—chronic kidney disease; PD—peritoneal dialysis; DMS—Dialysis Malnutrition Score; MIS—Malnutrition-Inflammation Score; IDWG—interdialytic weight gain. Data are presented as whole number (percentage), mean ± standard deviation or median (IQR). * chi-square test or *t*-test for independent samples. † post-dialysis stands only for the HD group. ‡ measured using body composition monitor.

**Table 2:** Laboratory parameters of the HD group and the control group.

Parameter	HD Group (N = 70)	Control Group (N = 60)	*p* *
Hemoglobin (g/L)	111.0 ± 9.9	127.6 ± 10.6	<0.001
Fasting glucose (mmol/L)	5.4 (4.7–6.9)	5.6 ± (5.0–6.2)	0.503
Pre-dialysis urea (mmol/L)	23.1 ± 5.6	-	-
Post-dialysis urea (mmol/L)	7.6 ± 3.1	5.7 ± 2.6 ^†^	<0.001
Pre-dialysis creatinine (μmol/L)	823.3 ± 191.8	-	-
Post-dialysis creatinine (μmol/L)	297.5 (245.0–376.0)	76.0 (71.0–85.0) ^†^	<0.001
Total bilirubin (μmol/L)	9.3 ± 3.0	10.8 ± 3.5	0.179
Total proteins (g/L)	65.8 ± 4.9	69.6 ± 4.2	<0.001
Albumins (g/L)	38.8 ± 3.0	40.4 ± 4.2	0.017
hsCRP (mg/L)	5.2 ± 3.6	1.4 ± 0.7	<0.001
Triglycerides (mmol/L)	1.9 ± 0.9	1.6 ± 0.8	0.108
Total cholesterol (mmol/L)	4.5 ± 0.9	4.42 ± 0.8	0.624
HDL (mmol/L)	1.0 ± 0.2	1.1 ± 0.2	0.005
LDL (mmol/L)	1.9 ± 0.9	1.6 ± 0.8	0.152

Abbreviations: hsCRP—high sensitivity C-reactive protein; HDL—high density lipoprotein; LDL—low density lipoprotein. Data are presented as mean ± standard deviation. * *t*-test for independent samples or Mann-Whitney U test. † post-dialysis stands only for the HD group.

**Table 3:** Correlation analysis between plasma adropin levels and biochemical, anthropometric, and clinical parameters in the HD group (N = 70).

Parameter	r * (*p*)	*p*
Total proteins (g/L)	−0.118	0.327
Albumins (g/L)	−0.131	0.278
Total bilirubin (μmol/L)	0.158	0.190
Fasting glucose (mmol/L)	−0.140 ^†^	0.247
hsCRP (mg/L)	−0.646	<0.001
Pre-dialysis systolic pressure (mmHg)	−0.301	0.011
Post-dialysis systolic pressure (mmHg)	−0.124	0.306
Pre-dialysis diastolic pressure (mmHg)	−0.299	0.011
Post-dialysis diastolic pressure (mmHg)	−0.184	0.127
Age (years)	0.109	0.366
Body mass index (kg/m^2^)	−0.234	0.051
Waist circumference (cm)	−0.141	0.242
Hip circumference (cm)	−0.058	0.631
CKD duration (years)	0.194 ^†^	0.106
DMS	−0.350 ^†^	0.003
MIS	−0.476 ^†^	<0.001
HD duration (years)	−0.305 ^†^	0.010

Abbreviations: HD—hemodialysis; CKD—chronic kidney disease; DMS—Dialysis Malnutrition Score; MIS—Malnutrition-Inflammation Score; hsCRP—high sensitivity C-reactive protein. * Pearson’s correlation coefficient. † Spearman’s rank correlation coefficient.

**Table 4:** Multiple linear regression model of independent predictors for serum adropin levels.

Variable	β *	SE ^†^	*t* Value	*p*
Age	−0.0006532	0.005	−0.123	0.902
Gender	−0.021	0.041	−0.517	0.607
BMI	−0.027	0.013	−2.006	0.049
HD duration	−0.031	0.009	−3.382	0.001
hsCRP	−0.058	0.019	−2.985	0.004
MIS	−0.310	0.140	−2.211	0.030
DMS	0.054	0.037	1.438	0.155
Body fat	−0.053	0.020	−2.631	0.010

Abbreviations: BMI—body mass index; hsCRP—high sensitivity C-reactive protein; HD—hemodialysis; DMS—Dialysis Malnutrition Score; MIS—Malnutrition-Inflammation Score. * unstandardized coefficient β. † standard error.

## Data Availability

All data is available with the corresponding author. You can contact him on email: josko.bozic@mefst.hr.
